# Supplementary material for: Chromothripsis during telomere crisis is independent of NHEJ, and consistent with a replicative origin
Source: Genome Res. 2019 May;29(5):737–49. doi: 10.1101/gr.240705.118 (PMC6499312; doi:10.1101/gr.240705.118)
Supplement: Supplemental Material [file supp_gr.240705.118_Supplemental_file_1.zip › contigs/annotated_contigs/DB51/contig.3.DB51_length_286_mean_cov_8.74825174825.docx]

**DB51_length_286_mean_cov_8.74825174825**

```
TCATGAGATCTGCTGATTTTACAAGTGTTTGATAGTTCCTCCTGTGTTCACTTCTCCTTCCTGCTGCCTTGTAAAGAGGGTGCCTTTCT
 >chr6:145790247-145790361 - E=3e-48
TCCCCTTTGCCTTCTGCCATAA|TTG|CTTCATATTTCTCATCAATACAAAACAGCTGAATACAAAAAGCAGCTGAGGCTCCCCTGTAG
                       >chr20:20144755-20144871 - E=2e-55
ATGGGGCGGAAGTGGACGGGCTCTTCAGGCAGTATGAGCGATGCCAAG|CTC|ATGATCCTTCCACCTCGGCCTCCCAAAGTGCTGGGA
                                                 >chr21:10171922-10171984 - E=5e-26
TTACAGGCATGAGCCACCACATC
```
